# Study on the changes of miRNAs and their target genes in regulating anthocyanin synthesis during purple discoloration of cauliflower curd under low temperature stress

**DOI:** 10.3389/fpls.2024.1460914

**Published:** 2024-12-03

**Authors:** Xingwei Yao, Qi Zhang, Haidong Chen, Xianhong Ge, Yangdong Guo, Daozong Chen

**Affiliations:** ^1^ Department of Vegetables, College of Horticulture, China Agricultural University, Beijing, China; ^2^ State Key Laboratory of Vegetable Biobreeding, Tianjin Academy of Agricultural Sciences, Tianjin, China; ^3^ College of Life Sciences, Ganzhou Key Laboratory of Greenhouse Vegetable, Gannan Normal University, Ganzhou, China; ^4^ National Key Laboratory of Crop Genetic Improvement, College of Plant Science and Technology, Huazhong Agricultural University, Wuhan, China

**Keywords:** cauliflower, cold stress, anthocyanins, miRNAs, degradome sequencing

## Abstract

**Introduction:**

Cauliflower is widely cultivated all over the world is attributed to its palatable flavor, high levels of anti-cancer compounds, and diverse array of nutrients. Exposure to extremely cold stress during production can result in a more frequent occurrence of purple discoloration in cauliflower curds. In response to cold stress, plants naturally produce anthocyanins to eliminate reactive oxygen species (ROS) generated as a defense mechanism.

**Methods:**

This research involved conducting mRNA sequencing analysis on cauliflower curds both before and after exposure to cold stress treatment.

**Results:**

It was determined that the up-regulation of anthocyanin biosynthesis-related genes *CHS, CHI, DFR, ANS, UGFT, PAP1/2*, and *MYBL2* occurred significantly in response to cold stress, resulting in a significant increase in total anthocyanin content. Subsequently, miRNA sequencing was employed to identify miRNAs in cauliflower curds, followed by differential expression analysis. The results showed that Bna-miR289 and Ath-miR157a may play a key role in regulating the accumulation of anthocyanin in cauliflower curds. Furthermore, we utilized degradome sequencing data to predict the target genes of the identified miRNAs, resulting in the identification of *BolK_3g48940.1, BolK_9g11680.1, BolK_7g41780.1, BolK_3g68050.1, *and *BolK_3g729700.1 *as targets. Subsequently, the expression patterns of the miRNAs and their target genes were validated using qRT-PCR, the results showed that Ath-miR157a and its target genes *BolK_3g68050.1 *and *BolK_3g72970.1* may be the key to the purple of cauliflower curds under cold stress.

**Discussion:**

Our preliminary findings identified key miRNAs and their target genes that may be involved in regulating anthocyanin synthesis, thereby enhancing the cold tolerance of cauliflower through mRNA, miRNA, and degradome sequencing. Overall, our study sheds light on the activation of anthocyanin synthesis in flower curds under cold stress conditions as a mechanism to enhance resilience to adverse environmental conditions.

## Introduction

Cauliflower, a cultivar of *Brassica oleracea* (CC, 2n=18) within the Brassicaceae family, is esteemed for its delectable flavor profile and crisp texture. Its enlarged inflorescence is a rich source of anticancer compounds and essential nutrients, making it a favored choice among consumers (Kapusta-Duch et al., 2012; Chen et al., 2024). Notably, cauliflower stands out as one of the rare vegetable varieties where the floral structures are consumed. However, the growth and development of cauliflower are particularly vulnerable to environmental influences compared to other edible plant parts such as leaves, fruits, and roots. In particular, cauliflower often faces challenges related to low temperature stress during both overwintering and spring cultivation periods. To effectively mitigate low temperature stress, cauliflower curds undergo synthesis and accumulation of anthocyanins, leading to a purple discoloration. However, surface buds of cauliflower exhibit a mottled purple hue due to insufficient and uneven distribution of anthocyanins, adversely impacting the marketability of the crop and posing increased risks for farmers (Rahim et al., 2019). The challenge of effectively addressing this issue in winter and spring cauliflower production remains unresolved. Hence, it is imperative for cauliflower breeding research to investigate the molecular mechanisms underlying the development of low temperature purple curds and to develop new varieties exhibiting this trait.

Plants have been documented to improve tolerance to abiotic stress through the synthesis and accumulation of anthocyanins across various species (Qin et al., 2021; Bhat et al., 2023; Sun et al., 2023; Guo et al., 2024). The biosynthetic pathway and transcriptional regulation mechanism of anthocyanins have been comprehensively elucidated (Jaakola, 2013; Tanaka et al., 2014; He et al., 2017; Sun et al., 2020; Zeng et al., 2023). In plants, genes encoding proteins involved in the anthocyanin biosynthetic pathway are categorized into early structural genes (EBGs) and late structural genes (LBGs) (Shi and Xie, 2014; Zhang et al., 2014). EBGs mainly include *PAL*, *C4H*, *4CL*, *CHS*, *CHI*, *F3H* (*F3’H*/*F3’5’H*), LBGs mainly include *DFR*, *ANS*, *UFGT*, *TT19* (Shi and Xie, 2014; Zhang et al., 2014). The transcriptional regulation of these structural genes plays a crucial role in their expression. Several transcription factors, including *MYB3*, *MYB4*, *MYB7*, *MYB32*, *MYB11*, *MYB12*, *MYB111*, *PAP1*, *PAP2*, *MYB113*, *MYB114*, *TT2*, *CPC*, and *MYBL2*, as well as bHLH transcription factors *TT8*, *GL3*, *EGL3*, and WD protein TTG1, play a crucial role in regulating the expression of key genes involved in the biosynthesis of phenylpropanoid compounds in plants. Specifically, *MYB3*, *MYB4*, *MYB7*, *MYB32* are involved in regulating *PAL*, *C4H*, and *4CL* expression, while *MYB11*, *MYB12*, *MYB111* are involved in regulating *CHS*, *CHI*, and *F3H* (*F3’H*/*F3’5’H*) expression (Zhao et al., 2013; Xu et al., 2014). The regulation of LBGs genes is intricate, with *PAP1*, *PAP2*, *MYB113*, *MYB114*, *TT2*, *CPC*, and *MYBL2* primarily influencing their expression (Zhao et al., 2013; Xu et al., 2014). Additionally, *PAP1*, *PAP2*, *MYB113*, MYB114, and *TT2* act as positive regulators of anthocyanin synthesis, while *CPC* and *MYBL2* act as inhibitors of anthocyanin biosynthesis in most plant species (Saigo et al., 2020; LaFountain and Yuan, 2021). In addition, more and more studies have shown that miRNAs are involved in the post-transcriptional regulation of plant anthocyanins (LaFountain and Yuan, 2021). miR828 and miR858 were first reported in *Arabidopsis thaliana* (Rajagopalan et al., 2006; Luo et al., 2012) were involved in the feedback loop regulation of anthocyanin, and then miR858 was found to inhibit the accumulation of anthocyanin in tomato (Jia et al., 2015).

The diversity of cabbage varieties is extensive, encompassing a wide range of colors in various tissues and organs including roots, stems, leaves, and inflorescences. In recent years, the advancement of sequencing technology has enabled the use of multi-omics joint analysis as a potent tool for investigating key plant traits. Prior research has utilized this approach to examine the color variations present in cabbage (*B. oleracea* var. *capitata*). Yan et al. (2019) employed bulked segregant analysis (BSA) by sequencing in conjunction with comparative transcriptome analysis to identify *BoMYB2* as a pivotal transcription factor governing anthocyanin biosynthesis in cabbage leaves, with various forms of variation within its promoter region serving as the primary contributors to color diversity across different subspecies. Similarly, Feng et al. (2021) utilized BSA sequencing alongside comparative transcriptome analysis to ascertain that *BoDFR1* plays a crucial role in promoting anthocyanin accumulation in pink leaf ornamental kale (*B. oleracea* var. *acephala*). Furthermore, Khusnutdinov et al. (2022) discovered that *MYBL2-1* exerts a negative regulatory influence on anthocyanin production in cabbage. Furthermore, researchers have shown a growing interest in the regulation at the post-transcriptional level. Chen et al. (2022b) employed a combination of transcriptome and degradome sequencing to systematically analyze miRNAs in pink ornamental kale, revealing that miR828 targets *Bo00835s060* to inhibit anthocyanin biosynthesis. Nevertheless, there is currently no literature available on the application of transcriptome and degradome sequencing to investigate the post-transcriptional regulation of miRNAs in cauliflower (*B. oleracea* L. var. *botrytis*) curd biosynthesis.

In this study, we sought to delve deeper into the molecular mechanism underlying the phenomenon of cauliflower curds turning purple in response to low temperature stress. To achieve this goal, we selected three groups of experimental materials with samples without cold stress (white), samples subjected to cold stress for 7 days (light purple), and for 21 days (purple) for transcriptome and degradation sequencing analyses. The study systematically identified and analyzed miRNAs in cauliflower curds, predicting their target genes through degradome sequencing. To validate the findings, qRT-PCR verification was conducted using white, light purple, and purple flower curds, Ath-miR157a and its target genes *BolK_3g68050.1* and *BolK_3g72970.1* may play a major role in regulating anthocyanin synthesis induced by cold stress in cauliflower curds. This research sheds light on the response of cauliflower curds to cold stress, specifically in relation to the synthesis and accumulation of anthocyanins, offering valuable insights into the transcriptional regulation mechanism of plant anthocyanins. Importantly, our results are of great value for analyzing the purple coloration mechanism of cauliflower, and are of great significance for the future development of molecular marker-assisted breeding.

## Materials and methods

### Plant materials

The experimental material NS13 utilized in this study demonstrated sensitivity to low-temperature stress. After 7 days of exposure to low temperatures, light purple coloration was observed in the flower buds on the surface of the cauliflower florets, with the intensity of the purple hue deepening as the duration of low-temperature treatment increased. To investigate the molecular mechanisms underlying NS13’s response to low temperature stress, white cauliflower curds, light purple cauliflower curds subjected to 7 days of low temperature stress, and purple cauliflower curds exposed to 21 days of low temperature stress were rapidly frozen in liquid nitrogen. Three biological replicates were collected for each treatment group and subsequently frozen in liquid nitrogen for two hours before being stored in a −80°C refrigerator. Surface bud samples of cauliflower curds representing three replicates of white, light purple, and purple were then submitted to Bioyi Biotechnology Co., Ltd. (Wuhan, China) for mRNA and small RNA sequencing. Then, DNBSEQ-T7 with PE150 model was used to mRNA sequencing, and NovaSeq 6000 platform (Illumina) platform was used to small RNA sequencing.

### Determination of total anthocyanin content in cauliflower curds

The synthesis and accumulation of anthocyanin in cauliflower curds following exposure to low temperatures is a common characteristic that may hinder the marketability of cauliflower. To investigate the variations in anthocyanin levels in cauliflower curds post low temperature stress, the total anthocyanin content was extracted from the surface buds of cauliflower curds in three replicates, each representing white, light purple, and purple varieties. The extraction method used in this study is detailed in Chen et al. (2022b).

### Differentially expressed genes in three periods of cauliflower curds through mRAN sequencing

The high-quality genome of cauliflower Korso was used as the reference genome for subsequent analysis (Guo et al., 2021). We used StringTie (v2.1.5) statistics to compare the Read Count values on each gene as the original expression of the gene, and then used FPKM to standardize the expression. Then difference expression of genes was analyzed by DESeq2 (v1.30.1) with screened conditions as follows: expression difference multiple |log2FoldChange| > 2, significant padj ≤ 0.01. Detailed mRNA sequencing expression levels, DEGs, Gene Ontology (GO) and Kyoto Encyclopedia of Genes and Genomes (KEGG) analysis methods refer to Cui et al. (2024).

### Small RNA sequencing data analysis

Small RNA sequencing reads were aligned to the reference genome using Bowtie, and the alignments of each sample as well as the distribution of the sRNA on the genome were counted. Small RNA sequencing and data analysis are basically the same as our previous methods (Chen et al., 2022b). The known miRNAs were identified through sequence alignment using the miRBase22.1 database (http://www.mirbase.org/index.shtml) and PmiREN database (Guo et al., 2022) without allowing mismatches. The determination of the differentially expressed miRNAs (DEMs) was made by applying a significance threshold of FDR < 0.05 and considering the absolute value of |Log2FC|≥1.

### Degradome sequencing and target gene identification

To conduct degradome sequencing, equal proportions of total RNA from white, light purple, and purple cauliflower curds surface buds were combined, and a sequencing library was prepared following the manufacturer’s instructions. Sequencing was carried out on the HiSeq 2500 platform at LC-BIO (Hangzhou, China) using a 50 bp single-ended read. Data analysis for degradome sequencing followed the methodology outlined in a prior publication by Chen et al. (2022b). To maintain data integrity, reads resembling sequences in Gen Bank and Rfam 14.1 were excluded. The remaining high-quality reads, ranging from 20 to 21 nucleotides in length, were subsequently aligned with the cDNA sequence of cauliflower reference Korso. The identification of potential cleavage sites was conducted using Cleveland pipeline version 4.4 through the analysis of identical sequences.

### Validation the expression patterns of miRNAs and their targets by qRT-PCR

To further investigate the mRNA and miRNA expression patterns associated with low temperature stress, total RNA was extracted from the surface buds of cauliflower curds in triplicate for white, light purple, and purple varieties, each sample with three biological replicates. The miRcute miRNA Isolation Kit (TIANGEN, Beijing, China) was utilized for the extraction of total RNA. The degradation and contamination of RNA was monitored by subjecting the sample to 1% agarose gel electrophoresis. We evaluated the RNA purity by utilizing the NanoPhotometer^®^ spectrophotometer (IMPLEN, CA, USA). The Qubit^®^ RNA Assay Kit was utilized in the Qubit^®^2.0 Flurometer (Life Technologies, CA, USA) to ascertain the RNA concentration. We assessed the RNA integrity by utilizing the RNA Nano 6000 Assay Kit from the Agilent Bioanalyzer 2100 system, manufactured by Agilent Technologies in CA, USA. To confirm the mRNA and miRNA expression, we performed quantitative real-time RT-PCR (qRT-PCR) validation on the key genes involved in the anthocyanin pathway and identified differentially expressed miRNAs. For mRNA quantification, SuperScript™II Reverse Transcriptase (Invitrogen, USA) and Oligo(dT) primers were utilized to synthesize first-strand cDNA. The Actin gene served as a control and gene-specific primers were designed (**Supplementary Table S7**). The poly (T) adaptor RT-PCR method described by Shi and Chiang (2005) was used for quantifying miRNA. In each sample, the TransScript miRNA First-Strand cDNA Synthesis SuperMix (TransGen, China) was used to reverse transcribe a total of 2µg of RNA into cDNA. The universal primer used was reversed, whereas the primers in the forward direction were distinct. The miRNA primers can be found in **Supplementary Table S7**. A Bio-Rad CFX96 Realtime System was utilized to conduct the qRT-PCR. For the qRT-PCR, three technical replicates were conducted using the SYBR^®^ Green PCR Supermix (CA, USA) as the chosen option. The reaction mixture consisted of 2μL of cDNA, 10μL of SYBR Supermix, 7.2μL of H2O, and 0.4μL of primer (10μM), with a total volume of 20μL. The reaction protocol consisted of heating at 98°C for 30 seconds, followed by 40 cycles of heating at 98°C for 10 seconds and cooling at 60°C for 30 seconds. The relative expression was determined using the 2−ΔΔCT approach. Detailed experimental and data analysis procedures can be found in the study by Chen et al. (2022a).

## Results

### Phenotypic characterization and analysis of total anthocyanin content of cauliflower curds

**Figure 1:**
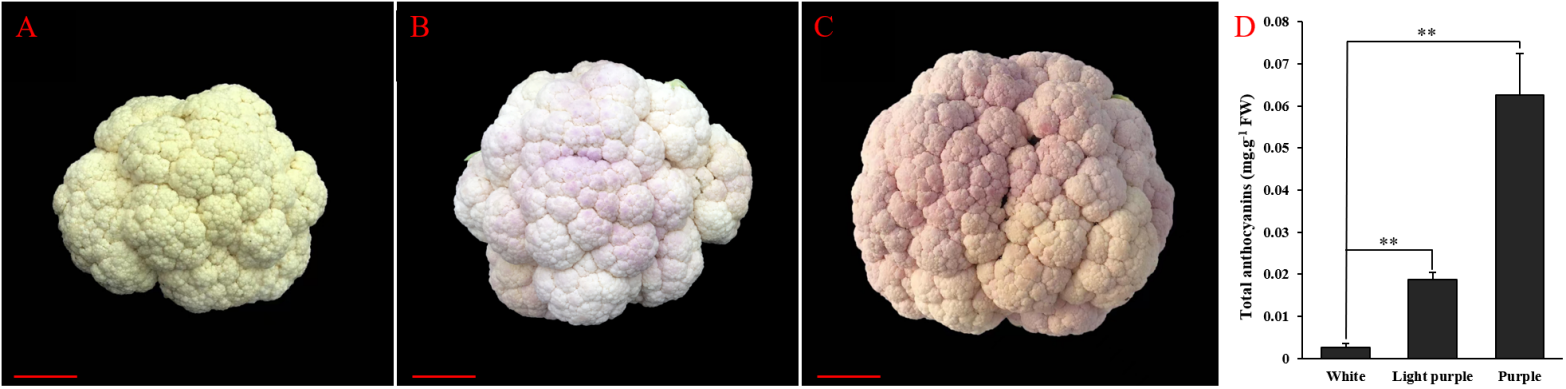
Phenotypic characterization and total anthocyanin content analysis of cauliflower curds. **(A)** Phenotype of white cauliflower curds; **(B)** Phenotype of light-purple cauliflower curds; **(C)** Phenotype of purple cauliflower curds; **(D)** Total anthocyanin content of white, light-purple and purple cauliflower curds. Both white, light-purple and purple samples are with six biological replicates, the point represents the mean value of three technical replicates in a representative biological experiment, the error bars indicate s.d, student’s t-test, **p<0.01.

In the context of field planting, mature cauliflower curds typically exhibit a white coloration (**Figure 1A**). In response to low temperature stress or chilling injury, cauliflower curds demonstrate an adaptive response by synthesizing and accumulating anthocyanins and other metabolites to enhance tolerance or resistance to adverse conditions. Consequently, the appearance of pale purple cauliflower florets is attributed to the accumulation of anthocyanins (**Figure 1B**). Moreover, prolonged exposure to low temperature stress or chilling injury results in a continued increase in anthocyanin synthesis and accumulation within cauliflower curds, leading to a gradual deepening of the purple coloration in the florets (**Figure 1C**). Concurrently, we assessed the anthocyanin levels in cauliflower florets during three stages: white, light-purple, and purple. The anthocyanin content in light-purple florets was notably greater than that in white florets, with a continued increase in anthocyanin content as stress persisted, ultimately leading to the development of a purple hue in the florets (**Figure 1D**). The uneven distribution of anthocyanins in cauliflower curds, resulting from varying degrees of stress on different parts of the vegetable, leads to the development of mottled purple discoloration. This ultimately diminishes the marketability of cauliflower curds, rendering them unsellable and causing significant economic losses for vegetable farmers.

### RNA-seq analysis of cauliflower curds in three periods

**Figure 2:**
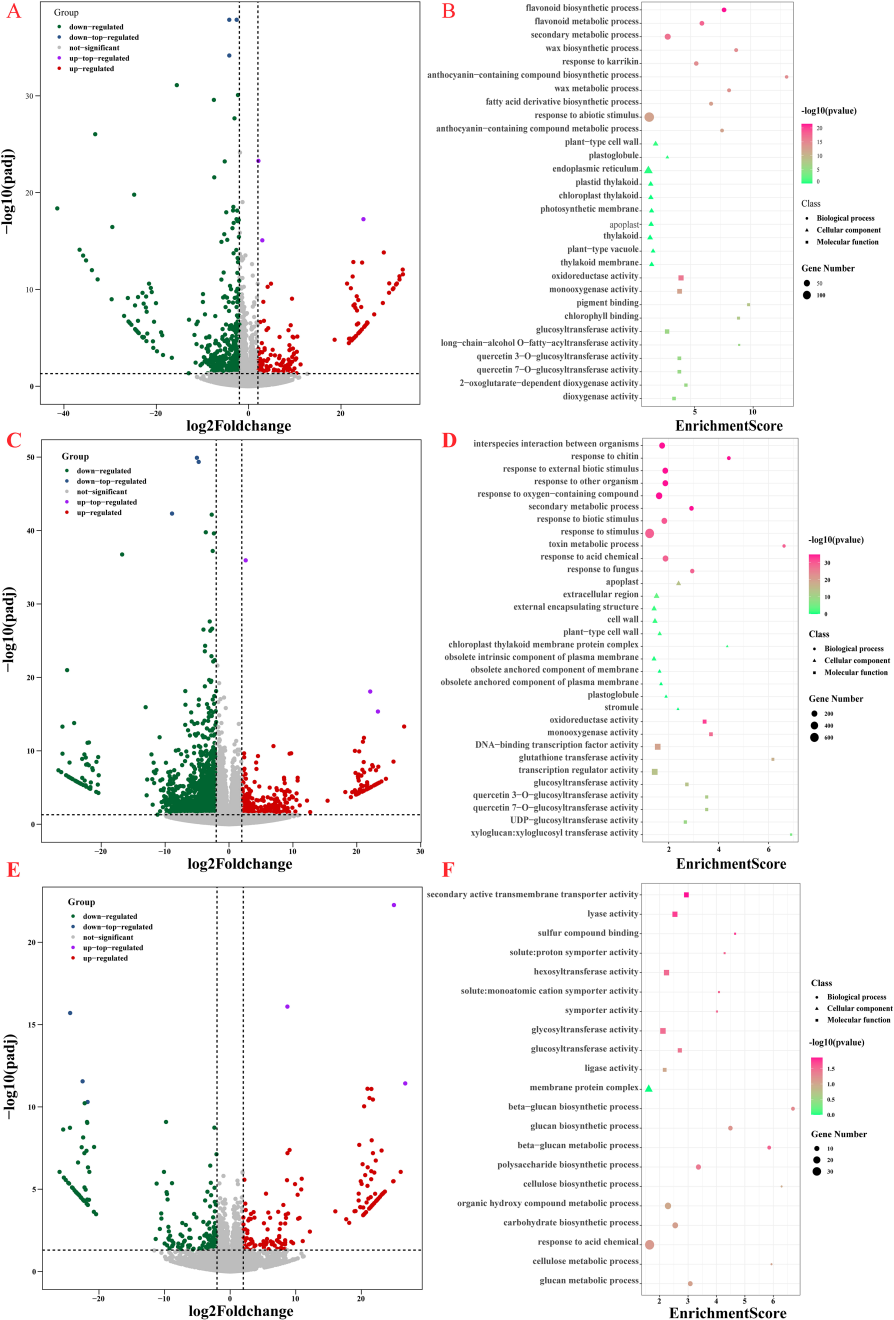
RNA-seq DEGs and GO enrichment analysis of cauliflower curds in three periods. **(A)** White and light-purple cauliflower curds DEGs volcano map; **(B)** GO enrichment analysis of DEGs in white and light-purple cauliflower curds; **(C)** White and purple cauliflower curds DEGs volcano map; **(D)** GO enrichment analysis of DEGs in white and purple cauliflower curds; **(E)** light-purple and purple cauliflower curds DEGs volcano map; **(F)** GO enrichment analysis of DEGs in light-purple and purple cauliflower curds.

To delve deeper into the molecular mechanisms underlying the response of cauliflower curds to low temperature or chilling stress, we conducted a comparative transcriptome analysis on untreated white cauliflower curds, light purple cauliflower curds subjected to 7 days of low temperature stress, and purple cauliflower curds exposed to 21 days of low temperature stress. Our findings revealed a total of 735 differentially expressed genes (DEGs) between light-purple and white cauliflower curds, with 533 DEGs showing up-regulation and 202 DEGs displaying down-regulation. A total of 1917 DEGs were identified between purple and white cauliflower curds, comprising 1521 up-regulated DEGs and 396 down-regulated DEGs. Additionally, 360 DEGs were found between light-purple and purple cauliflower curds, with 198 up-regulated DEGs and 162 down-regulated DEGs (**Figures 2A, C, E**; **Supplementary Table 1**). To elucidate the functional implications of these DEGs, Gene Ontology (GO) and Kyoto Encyclopedia of Genes and Genomes (KEGG) enrichment analyses were conducted. The DEGs identified between light purple and white cauliflower curds exhibited enrichment of genes primarily involved in the response to abiotic stimuli pathway, with the highest p-value observed in the flavonoid biosynthetic process and the secondary metabolic process. Additionally, significant enrichment was observed in the anthocyanin-containing compound biosynthetic process and anthocyanin-containing compound metabolic process (**Figure 2B**). In contrast, the DEGs between purple and white cauliflower curds enriched genes primarily associated with the response to stimulus pathway, oxidoreductase activity, interspecies interaction between organisms, and response to chitin, response to external biotic stimulus. Non-biological stress response-related pathways, such as those involved in response to other organisms and oxygen-containing compounds, exhibited significant enrichment with higher p-values and Enrichment scores within the secondary metabolic process category (**Figure 2D**). Notably, differentially expressed genes between light purple and purple cauliflower curds displayed the greatest enrichment in response to organic substances, along with the highest p-values in pathways related to secondary active transmembrane transporter activity, lyase activity, and other processes (**Figure 2F**). These findings indicate that during the initial phase of exposure to low temperature stress, cauliflower curds exhibit a rapid response to this stress, resulting in a high enrichment of genes associated with the response to stimuli. Additionally, the initiation of anthocyanin biosynthesis serves as a mechanism to combat adversity, leading to a significant enrichment of items related to anthocyanin biosynthesis and metabolic pathways, such as the biosynthetic process of anthocyanin-containing compounds. After a three-week period of stress, a significant accumulation of anthocyanin was observed in the surface buds of cauliflower curds, resulting in the enrichment of numerous genes in six pathways, including oxidoreductase activity, to enhance tolerance and resistance to low temperature stress. Additionally, only the secondary metabolic process was enriched within secondary metabolic pathways.

### Expression patterns of genes related to anthocyanin biosynthesis pathway in cauliflower curds at three stages

**Figure 3:**
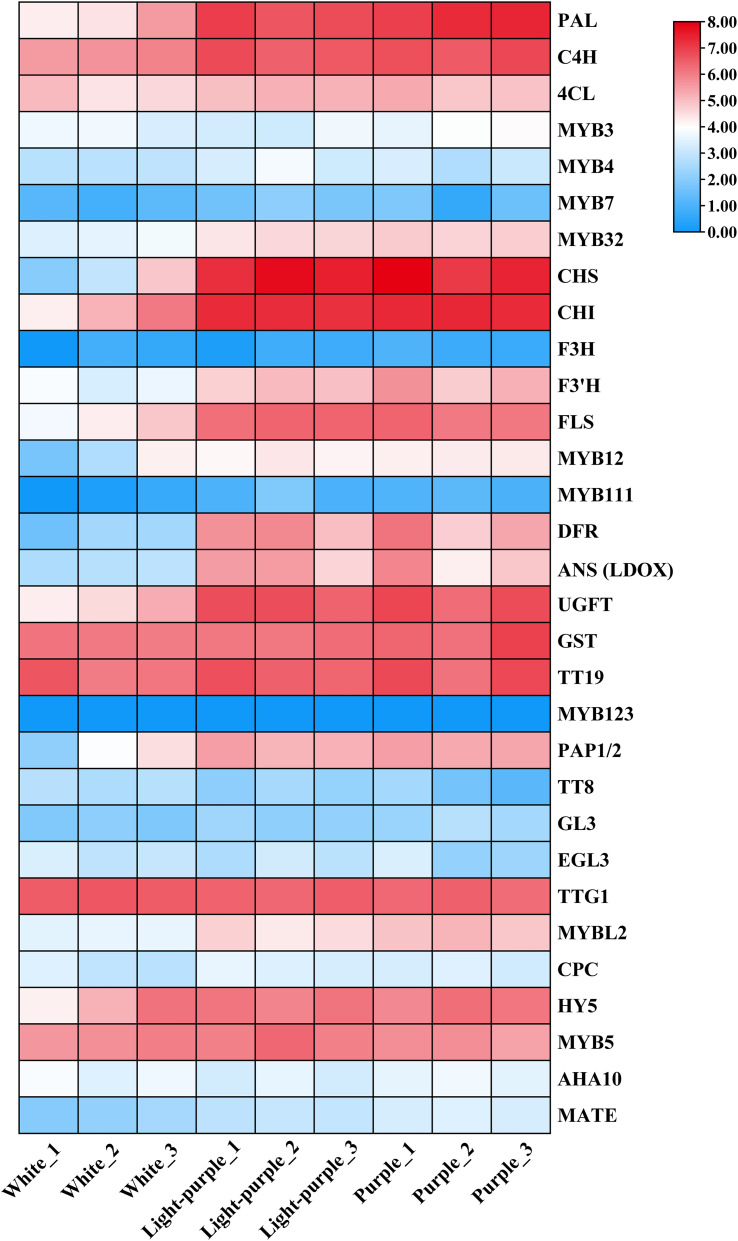
The expression patterns in white, light-purple and purple cauliflower curds were plotted using the FPKM values of anthocyanin biosynthetic pathway-related genes. The FPKM values of all samples were taken log2 logarithm and used for plotting, and the colors on the heat map, from blue to white and red, indicate the expression level from low to high.

Cauliflower curds demonstrate the ability to withstand stress through the activation of anthocyanin biosynthesis and accumulation following exposure to low temperatures. To investigate the expression profiles of genes related to the anthocyanin biosynthetic pathway during various stages of low temperature stress, we utilized the protein sequences of 31 such genes identified in *Arabidopsis thaliana* as reference sequences, leading to the discovery of 140 anthocyanin biosynthetic pathway-related genes within the entire genome of cauliflower. The FPKM values for the genes in question were derived from transcriptome sequencing data of cauliflower curds at three developmental stages (**Supplementary Table 2**). Subsequently, these FPKM values were utilized to generate an expression heat map for the three stages of cauliflower curds, as illustrated in **Figure 3**. Within the phenylpropanoid synthesis pathway, the structural genes *PAL1*, *C4H*, and *4CL* exhibited significant up-regulation in light-purple and purple cauliflower curds, while only the transcriptional regulator *MYB32* showed a significant increase in expression level. In the flavonoid biosynthesis pathway, the expression levels of structural genes *CHS*, *CHI*, *F3’H*, and *FLS* were notably up-regulated in light-purple and purple cauliflower curds, with *CHS* and *CHI* showing particularly significant increases. The expression of *MYB12* exhibited a consistent trend with that of the structural genes. In the anthocyanin synthesis pathway, the expression levels of structural genes *DFR*, *ANS* (*LDOX*), *ANR*, *UGFT*, *GST*, and *TT19* were markedly up-regulated in light-purple and purple cauliflower curds, while the expression patterns of transcription regulators *PAP1/2* and *MYBL2* were consistent with the expression patterns of structural genes. Specifically, the genes *DFR*, *ANS* (*LDOX*), and the transcription factor *PAP1/2* exhibit specific expression patterns in light-purple and cauliflower curds, with minimal expression in white cauliflower curds. This differential expression may play a crucial role in the synthesis and accumulation of anthocyanins in these tissues. Additionally, previous research has shown that *MYBL2* acts as an inhibitor in the anthocyanin pathway, and its expression profile aligns with that of *DFR*, *ANS* (*LDOX*), and *PAP1/2*.

### Identification of miRNAs in cauliflower

**Figure 4:**
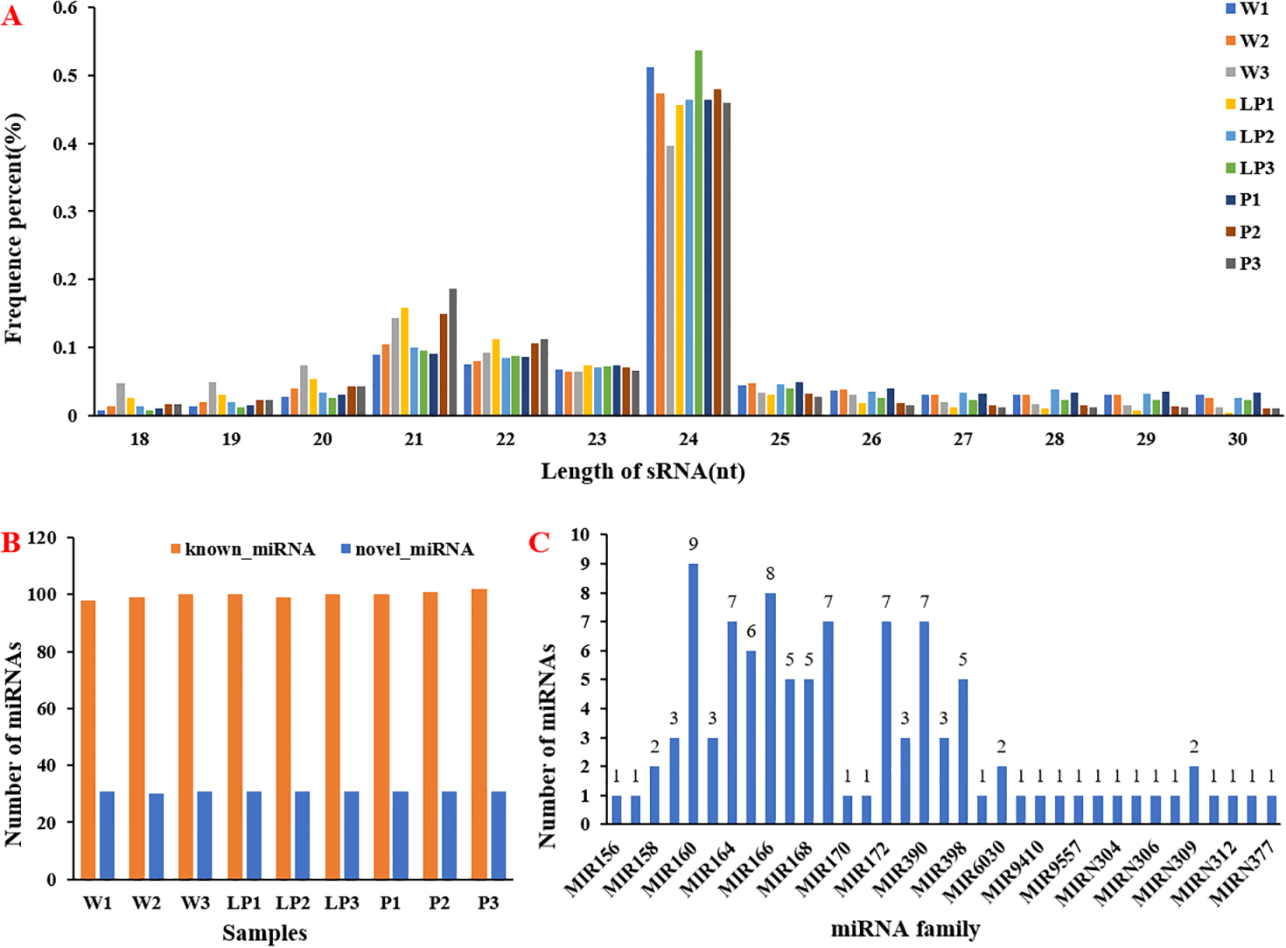
Identification and analysis of miRNAs. **(A)** Length distribution of sRNAs; **(B)** The number of miRNA in 9 samples; **(C)** Distribution of miRNA families.

To further elucidate the post-transcriptional regulation mechanism of cauliflower curds in response to stress, a systematic identification of miRNAs was conducted in cauliflower, resulting in the identification of 431 miRNAs (**Supplementary Table 3**). The findings indicated that 24nt miRNAs were the most prevalent among the 18-30nt long miRNAs, with 21-23nt miRNAs following closely behind. However, the distribution of miRNAs of varying lengths varied among white, light purple, and purple cauliflower curds. For instance, LP3 exhibited the highest proportion of 24nt miRNAs, while P3 showed the highest proportion of 21nt long miRNAs (**Figure 4A**). Subsequently, we classified the identified miRNAs into known-miRNAs and noval-miRNAs. The distribution of known-miRNAs and noval-miRNAs in white, light purple and purple curds9 group materials was relatively uniform. There were about 100 known-miRNAs and about 30 noval-miRNAs (**Figure 4B**). Further, we classified the identified known-miRNAs according to the miRNA family. Among the identified 35 known-miRNA families, 18 miRNA families identified only one family member, 3 miRNA families identified two family members, 4 miRNA families identified three family members, 3 miRNA families identified five family members, 1 miRNA family identified six family members, and 4 miRNA families identified seven family members. One miRNA family was identified to 8 family members, and the most miRNA family was MIR160, a total of 9 family members were identified (**Figure 4C**). To further investigate the key miRNAs responsive to and resilient against stress during the white, light-purple, and purple stages, we conducted an analysis of differentially expressed miRNAs within these groups. A total of 21 differentially expressed miRNAs (DEMs) were identified between the white and light-purple stages, with 9 exhibiting up-regulation and 12 showing down-regulation. Similarly, 30 DEMs were observed between the white and purple stages, with 2 being up-regulated and 28 being down-regulated (**Supplementary Table 4**).

### Identification of miRNAs target genes based on degradome sequencing

**Figure 5:**
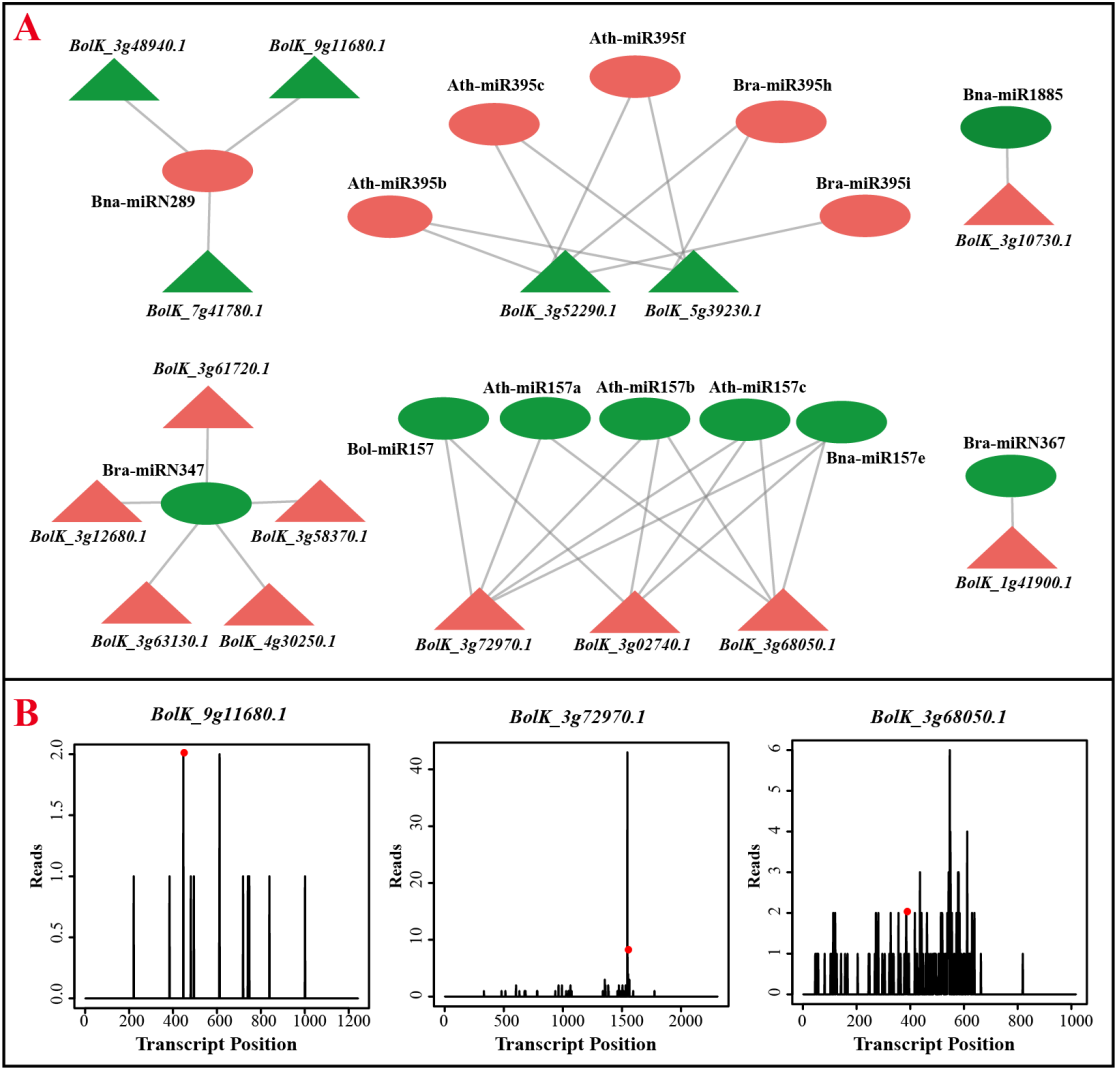
Negatively regulated miRNAs and their target genes. **(A)** Negatively regulated miRNA-target interaction pairs. Circles represent miRNAs and triangles represented the target genes, red and green represented of up and down expressed in white curds, respectively. **(B)** The target genes of Bna-miRN289 and Ath-miR157a, the t-plots confirmed by degradome sequencing.

MiRNAs function by targeting specific genes and cleaving their transcripts, leading to impaired gene function. To analyze the target genes of identified miRNAs, degradome sequencing was conducted using young cauliflower curds. A total of 2949 target genes were predicted for 431 miRNAs, with most miRNAs targeting multiple transcripts (**Supplementary Table 5**). The number of target genes per miRNA ranged from 1 to 15, with an average of approximately 6. Simultaneously, alongside the phenomenon of one microRNA regulating multiple transcripts, certain microRNAs also target a common gene. To delve deeper into the roles of Differentially Expressed MicroRNAs (DEMs) and their target genes in the context of responding to and combating adversity, we specifically chose several instances where a microRNA exhibited upregulated expression while its target gene showed downregulated expression, as well as cases where a microRNA displayed downregulated expression while its target gene exhibited upregulated expression. These selected combinations are depicted in **Figure 5**. The diagram illustrates the complex interplay between miRNAs and target genes, showing that a single miRNA can target multiple genes, and multiple miRNAs can target a single gene, resulting in an intricate targeting network. For instance, the up-regulation expression of Bna-miRN289 in cauliflower curds at various stages resulted in the down-regulation expression of target genes *BolK_3g48940.1*, *BolK_7g41780.1*, and *BolK_9g11680.1*. Similarly, the down-regulation of Bra-miRN347 resulted in the up-regulation of its target genes *BolK_3g61720.1*, *BolK_3g58370.1*, and *BolK_3g12680.1*. The expression patterns of *BolK_3g63130.1* and *BolK_4g30250.1* exhibited contrasting trends (**Figure 5A**; **Supplementary Tables 5**, **6**). Furthermore, the degradation categories of various target genes in the degradation group sequencing varied, with examples including *BolK_9g11680.1* category=1, *BolK_3g72970.1* category=2, and *BolK_3g68050.1* category=3 (**Figure 5B**). These distinct category values are associated with the level of targeting precision, ultimately influencing the efficacy of miRNA-mediated cleavage of target genes.

### RT-PCR analysis of miRNAs and their target genes in white, light purple and purple cauliflower curds

**Figure 6:**
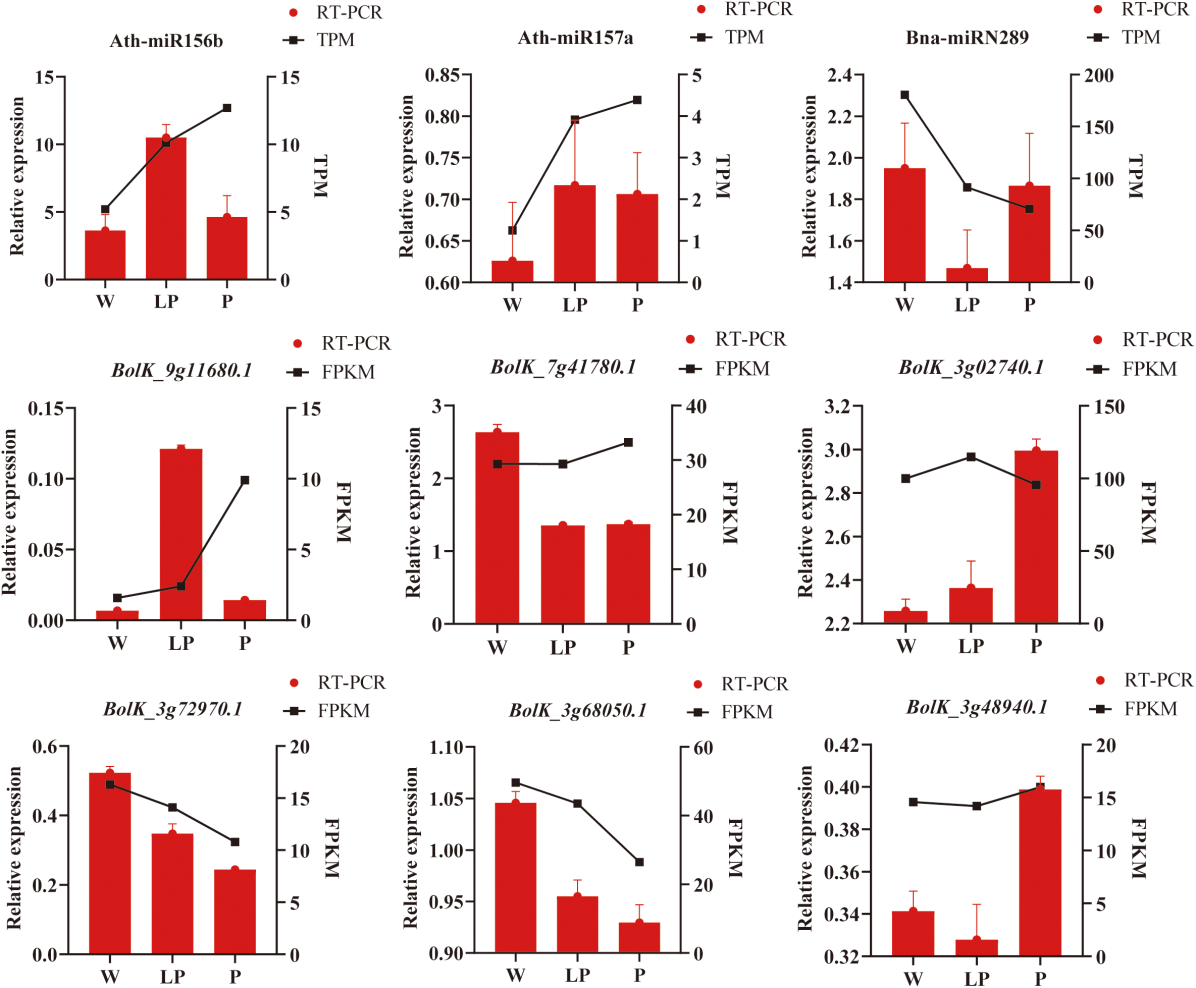
qRT-PCR validation of miRNAs and target genes. The red column represents the results of qRT-PCR verification, and the black line represents the expression level of transcriptome sequencing analysis.

In order to corroborate the findings of transcriptome sequencing and degradome sequencing analyses, we conducted qRT-PCR validation using white, light purple, and purple cauliflower curds. The results indicated that the overall expression patterns of 3 miRNAs and 6 target genes were in agreement with the transcriptome sequencing results (**Figure 6**). Specifically, Ath-miR156b and Ath-miR157a exhibited decreased expression in white cauliflower curds and increased expression in cauliflower curds with anthocyanin synthesis, while Bna-miRN289 displayed the highest expression level in white cauliflower curds. Of the six target genes examined, *BolK_3g48940.1* exhibited the highest expression level in purple cauliflower curds and the lowest in light purple cauliflower curds. The expression level of *BolK_3g02740.1* displayed a positive correlation with the intensity of cauliflower curds color. Conversely, the expression levels of *BolK_3g68050.1* and *BolK_3g72970.1* demonstrated a consistent downward trend as the cauliflower curds color deepened. *BolK_9g11680.1* exhibited the highest level of expression in light purple cauliflower curds, while displaying the lowest level of expression in white cauliflower curds. Conversely, the expression level of *BolK_7g41780.1* was highest in white cauliflower curds, gradually decreasing in light purple and purple varieties. In summary, the results of expression pattern analysis and qRT-PCR verification showed that the expression level of Ath-miR157 an increased with the deepening of cauliflower curds color, while the expression levels of its target genes *BolK_3g68050.1* and *BolK_3g72970.1* were correspondingly down-regulated, which may be the key to regulate cauliflower curds anthocyanins biosynthesis and accumulation under cold stress.

## Discussion

*B. oleracea* encompasses numerous subspecies, including cabbage, Chinese kale, ornamental kale, kohlrabi, cauliflower, and botrytis, each exhibiting diverse tissue and organ variations in colors such as green, white, and purple (Yan et al., 2019). Cauliflower and botrytis are prominent examples of inflorescence mutation resulting in the formation of flower heads, with cauliflower typically displaying white curds and botrytis showcasing green curds, and these two subspecies are extensively cultivated globally due to their abundant nutritional benefits (Kapusta-Duch et al., 2012). As a result of the ongoing degradation of the global climate, the prevalence of abiotic stress has escalated worldwide, impacting plants cultivated in agricultural fields (Naing and Kim, 2021). One such stressor, low temperature stress, induces the purple discoloration of cauliflower and botrytis curds through the synthesis and accumulation of anthocyanin, a phenomenon frequently observed in field production. Researchers have conducted studies to address this industrial issue (Rahim et al., 2019; Liu et al., 2020; Chen et al., 2022a).

### The synthesis and accumulation of anthocyanin is beneficial to the tolerance of cauliflower to cold stress

Plants exhibit an increase in reactive oxygen species (ROS) production following exposure to cold stress, which in turn triggers the activation of stress resistance mechanisms. However, under severe stress conditions, excessive ROS production can lead to oxidative damage in plants. In response to this, plants regulate ROS levels through the synthesis and accumulation of anthocyanins for antioxidant activity, thereby promoting sustainability. In the present study, we conducted comparative transcriptome sequencing and analysis of anthocyanin content in white cauliflower curds prior to cold stress and light purple cauliflower curds following 7 days of cold stress, and purple cauliflower curds after 21 days of cold stress. The findings indicate that following a 7-day cold treatment, DEGs in cauliflower were predominantly enriched in the response to abiotic stimuli, suggesting a rapid initiation of response to cold stress. Specifically, DEGs exhibited the highest statistical significance in the flavonoid biosynthetic process and the greatest enrichment in the anthocyanin-containing compound biosynthetic process. These results suggest that the biosynthesis and accumulation of anthocyanin in cauliflower curds play a significant role in enhancing chilling tolerance as a response to abiotic stimuli. Following 21 days of cold stress, DEGs exhibited the greatest enrichment in the response to stimulus category. Additionally, the interspecies interaction between organism category displayed the highest p-value, and the toxin metabolic process category demonstrated the highest enrichment index. These findings suggest that the cold stress tolerance of cauliflower curds was augmented through the accumulation of anthocyanin after the 21-day cold treatment, leading to a coordinated metabolism of toxins produced in response to the cold stress within the tissues. These results are consistent with previous reports that plant synthesis and accumulation of anthocyanins can improve tolerance to abiotic stresses (Ahmed et al., 2015; Naing and Kim, 2021; Li and Ahammed, 2023).

### Cauliflower tolerance to cold stress has multiple levels of regulation

The main mechanism by which miRNAs regulate the transcription of target genes is to target the cleavage of target genes and cause their loss of function. For example, in Arabidopsis and tomato, miR828 and miR858 inhibit anthocyanin accumulation by targeting *PAP1* and its homologues. This study utilized a comprehensive approach involving mRNA, miRNA, and degradome sequencing to investigate the molecular mechanisms underlying the response and tolerance of cauliflower curds to cold stress. Three significant miRNAs were discovered in white, light-purple, and purple cauliflower curds, and their respective target genes were identified through degradome sequencing. Notably, Bna-miRN289 exhibited the highest expression level in white cauliflower curds, with its expression decreasing upon exposure to cold stress. The expression patterns of the target genes *BolK_3g48940.1*, *BolK_9g11680.1*, and *BolK_7g41780.1* exhibited contrasting trends to those of Bna-miRN289, which showed up-regulation in tissues undergoing anthocyanin synthesis and accumulation. *BolK_9g11680.1* displayed the highest expression level in light purple flower curds, while *BolK_3g48940.1* exhibited the highest expression in purple cauliflower curds. Importantly, the expression pattern of Ath-miR157a was opposite to that of Bna-miRN289, with Ath-miR157a showing the lowest expression in white cauliflower curds and an up-regulation in expression level with the progression of cold stress. The expression profiles of the target genes *BolK_3g68050.1* and *BolK_3g72970.1* exhibited inverse patterns compared to Ath-miR157a. The highest expression levels were observed in white cauliflower curds, with a subsequent down-regulation as anthocyanin biosynthesis and accumulation progressed. The mechanism of miRNA action involves targeted binding to specific genes for cleavage, thereby inhibiting their normal function. Thus, the results indicated that Ath-miR157a positively regulates the synthesis and accumulation of anthocyanins in cauliflower, leading to the development of purple cauliflower curds that exhibit responsiveness to and tolerance of cold stress, whereas Bna-miRN289 may serve as a negative regulator. Chowdhury et al. (2021) conducted a comprehensive analysis of miRNAs and lncRNAs in cauliflower, predicting their roles in post-transcriptional gene regulation at a genome-wide level. In the study conducted by Chen et al. (2022b), it was discovered that miR828 modulates anthocyanin production in ornamental kale through its interaction with *BoPAP1*. Our results preliminarily indicated that Ath-miR157a targeted cleavage of *BolK_3g68050.1* and *BolK_3g72970.1*, which increases the synthesis and accumulation of anthocyanins, thereby transforming cauliflower curds from white to purple. In *Ammopiptanthus nanus*, miR530 targeting *TZP* may be the key to enhance its cold tolerance (Zhu et al., 2023). Our study found for the first time that Ath-miR157a targets *BolK_3g68050.1* and *BolK_3g72970.1* to positive regulate the accumulation of anthocyanins and enhance the cold tolerance of cauliflower florets. However, due to the differences in the regulation of cold tolerance in different plants or different tissues of plants, our results need to be further verified by subsequent experiments. Furthermore, our ongoing research will investigate the regulatory mechanisms between the identified miRNAs and their target genes. We aim to develop molecular marker-assisted breeding techniques and integrate gene editing technology to produce cauliflower lines with enhanced cold resistance, thereby facilitating the breeding of new varieties of high-quality, cold-resistant cauliflower.

## Data Availability

The mRNA and microRNA sequencing data of this research have been deposited in the NCBI (National Center for Biotechnology Information) database with biological projects PRJNA1127765.
